# Prevalence and characteristics of Postpartum Depression symptomatology among Canadian women: a cross-sectional study

**DOI:** 10.1186/1471-2458-11-302

**Published:** 2011-05-11

**Authors:** Andrea Lanes, Jennifer L Kuk, Hala Tamim

**Affiliations:** 1 Kinesiology & Health Science, York University, Toronto, Ontario, Canada

## Abstract

**Background:**

This study aims to look at the prevalence and characteristics of postpartum depression symptomatology (PPDS) among Canadian women. Studies have found that in developed countries, 10-15% of new mothers were affected by major postpartum depression. Mothers who suffer from postpartum depression may endure difficulties regarding their ability to cope with life events, as well as negative clinical implications for maternal-infant attachment.

**Methods:**

An analysis based on 6,421 Canadian women, who had a live birth between 2005 and 2006 and were part of the Maternity Experience Survey (MES), was performed. PPDS was measured based on the Edinburgh Postnatal Depression Scale. Various factors that assessed socio-economic status, demographic factors, and maternal characteristics were considered for the multinomial regression model.

**Results:**

The national prevalence of minor/major and major PPDS was found to be 8.46% and 8.69% respectively. A mother's stress level during pregnancy, the availability of support after pregnancy, and a prior diagnosis of depression were the characteristics that had the strongest significant association with the development of PPDS.

**Conclusions:**

A significant number of Canadian women experience symptoms of postpartum depression. Findings from this study may be useful to increase both the attainment of treatment and the rate at which it can be obtained among new mothers. Interventions should target those with the greatest risk of experiencing PPDS, specifically immigrant and adolescent mothers.

## Background

Postpartum depression (PPD) refers to a non-psychotic depressive episode that begins in or extends into the postpartum period [1,2]. PPD can evolve from a preexisting case of the baby blues, or can become apparent after the first weeks of giving birth and can last as long as 14 months [1,3-5]. Symptoms include anxiety, guilt, negative maternal attitudes, and poor parenting self-efficacy [3,6,7]. A multitude of treatment options for PPD exist, including interpersonal therapy and the most common treatment, pharmaceutical intervention [1,8].

Mothers who suffer from PPD endure significant consequences, especially with their ability to cope with life events, including parenting tasks [9]. PPD elicits negative clinical implications for maternal-infant attachment; there is a withdrawn and disengaged behaviour in the mother and/or intrusive and hostile mother-infant communication [10-13]. Research has shown that experiencing symptoms of PPD can have immediate ill effects on the offspring [14].

PPD is a major health concern for women from diverse cultures [15]. Internationally, the prevalence of major PPD ranges from almost 0% in Singapore to nearly 57% in Brazil [12]. In 1998, Statistics Canada reported that in Canada, 10-15% of new mothers were affected by major PPD, which is similar to prevalence rates found in other developed countries [1,6]. However, there are no recent studies that show the overall provincial and national prevalence rates of PPD, and the few studies that have been performed show varying results, refer to specific communities that may not be representative of the Canadian population. For example, the prevalence of PPD was found to range from 4.3 to 15.2% in postpartum patients from five Ontario hospitals [16]. Similarly, in a study utilizing the EPDS, Davey et al. (2008) found that 6.5% of a cohort of medically low risk pregnant women in the Calgary Health Region displayed symptoms of minor/major PPD while 4.5% scored in the range of major PPD [6].

The range of international PPD prevalence rates may be due to cross-cultural variables, screening methods, differences in the perception of mental health and its stigma, or differences in socio-economic backgrounds [12]. Globally, predictors of PPD vary as well, however usually four main categories emerge; socio-economic, demographic, maternal and social support. Total household income has been negatively associated with the prevalence of PPD in women in cohorts in Vancouver and Calgary, Canada [6,17]. Conversely, the risk for PPD symptomatology increased when the mother was born outside of Canada, even after adjusting for socioeconomic status [6]. Maternal characteristics that influence PPD include prior experience of depressive episodes preceding pregnancy, which has consistently been linked with the occurrence of PPD in mothers [6,18,19]. Previous pregnancy experience, as well as the development of a new medical condition during pregnancy has been positively associated with the onset of PPD [20,21]. Additionally, marital stress and the lack of prenatal care have both been associated with a higher risk of PPD symptomatology [17,18,21]. Studies have found that the presence of low levels of social support have been linked to the development of PPD [1,6].

Given the implications of PPD on the mother and the child, knowledge of prevalence rates and predictors are necessary to implement preventative measures and aid health practitioners in addressing at-risk groups. Few studies have been conducted examining Canadian PPD prevalence rates and predictors, and these studies have been limited to specific populations. Hence, national and provincial Canadian estimates of PPD from a single study are limited. This study aims to assess the current national and provincial prevalence rates of PPD symptomatology and the characteristics of PPD among Canadian women 15 years of age or greater.

## Methods

The analysis of this study was based on data from the Maternity Experience Survey (MES), which was designed by the Maternity Experiences Study Group of the Canadian Perinatal Surveillance System, and sponsored by the Public Health Agency of Canada. The survey was conducted by Statistics Canada between October 23, 2006 and January 31, 2007. The MES is the first nationwide survey that assessed pregnancy, delivery and postnatal experiences of mothers and their children. The survey sample was selected from the Canadian Census of Population to include women aged 15 years and above who had singleton live births between the period of February 15, 2006 and May 15, 2006 in the provinces of Canada and between November 1, 2005 and February 1, 2006 in the territories of Canada. A total of 8,542 Canadian women were selected, out of which 6,421 responded to the survey. In the provinces, computer assisted telephone interviews were used for data collection, whereas in the territories, a personal interview with a paper version of the questionnaire was offered, if a telephone interview was not possible. The telephone interview lasted on average 45 minutes. The MES has been previously described in previous reports [22]. Privilege to use the MES was granted by Statistics Canada Research Data Centre. Additionally, screening and ethics were required from the Research Data Centre.

The main outcome of the study was defined as: postpartum depression symptomatology (PPDS) assessed by the score on the Edinburgh Postnatal Depression Scale (EPDS). The EPDS is a comprehensive and widely used screening tool for detecting symptoms of PPD [23]. In developed countries a score of 0-9 inclusively indicates no risk of experiencing symptoms of PPD; a score of 10-12 indicates a minor/major risk of experiencing symptoms of PPD; and a score of 13 or greater indicates a major risk of experiencing symptoms of PPD [8,23]. The sensitivity and specificity of the EPDS has been found to be 75% and 84% respectively, at a cut-off of 13 [24], and has been used and found to be valid in Canadian studies [8,25,26]. Major depression is defined as a clinical syndrome that has a clinical treatment process. Symptoms can include, but are not limited to, anxiety, sleep disorders, a sense of detachment from infant, irritability, and fatigue. Minor/major depression is a lesser form of major depression, where early detection and treatment can prevent further exacerbation of symptoms [7,23]. A wide range of independent variables were investigated as potential predictors of PPDS. Socio-economic factors, such as maternal education level (less than high school, high school graduate, some post-secondary, post-secondary diploma, university graduate), household income (low, low-middle, middle, upper-middle, high), and occupation during pregnancy and demographic factors, consisting of region of residence (Atlantic, Quebec, Ontario, Prairies, British Columbia, Territories), immigration status, and the age of the infant (5 months, 6-8 months, 9-14 months) were examined. Information about maternal characteristics including parity, living with a husband/partner, maternal age at time of interview (15-19, 20-24, 25-34, 35-39, 40+), pregnancy weight gain guidelines (inadequate, recommended, excessive), previous diagnosis of depression/prescription antidepressants, smoking status during 3^rd ^trimester of pregnancy, mother's stress level during pregnancy (very, somewhat, not), and breastfeeding initiation were explored. Finally, other variables, availability of support after pregnancy (none of the time, some of the time, most of the time), planned pregnancy, and baby in Neonatal Intensive Care Unit (NICU) were analyzed. All the variables were directly self-reported by the mother. Household income was calculated based on the number of people in the household and the total household income before taxes and deductions earned by all household members from all sources in the past 12 months [27]. The pregnancy weight gain guidelines were based on the Institute of Medicine's recommendations for total weight gain during pregnancy [28]. A mother's stress level during pregnancy was based on the amount of stress reported during the 12 months prior to the baby's birth [29]. Support was defined as companionship, assistance and any other type of support that may have been required [29].

The prevalence of PPDS was estimated using population weights and examined across all the Canadian provinces and territories. At the univariate level, differences in the proportion of PPDS were assessed among the different levels of each predictor using population weights. Odds ratios (OR) with 95% confidence intervals (95% CI) were calculated. All the independent variables were considered in a multinomial multivariate logistic regression analysis where the dependent variable was PPDS with the referent category being not having PPDS. Adjusted OR and 95% CI were reported for the final model. To account for the complex sampling design, bootstrapping was performed to calculate all the 95% CI estimates [30]. All analyses, in exception to bootstrapping, were conducted using the Statistical Package for Social Sciences (SPSS, version 17.0). Bootstrapping was performed using the Stata: Data Analysis and Statistical Software (Stata, version 10.1).

## Results

**Figure 1:**
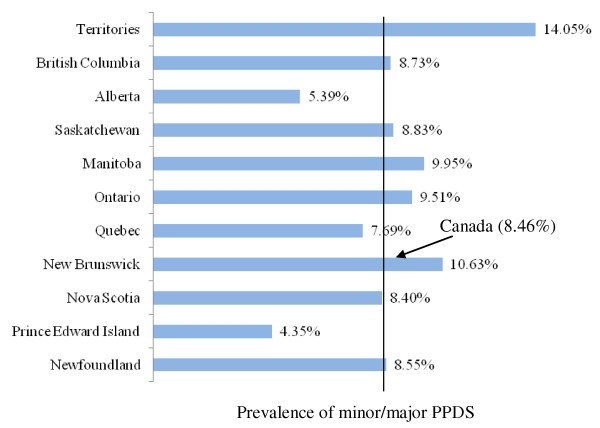
Distribution of minor/major PPDS across Canadian provinces and territories

**Figure 2:**
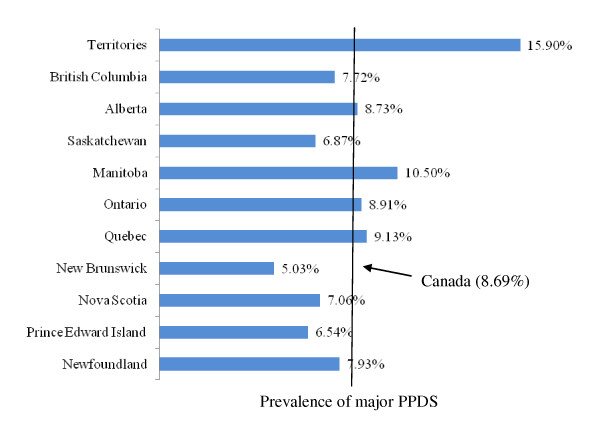
Distribution of major PPDS across Canadian provinces and territories

**Figure 3:**
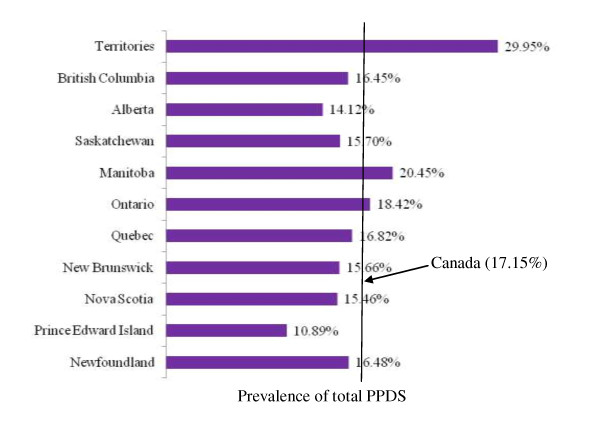
Distribution of total PPDS (minor/major & major) across Canadian provinces and territories

For the present study the sample size analyzed was 6,421 weighted to represent 76,509 Canadian women. Figure 1 and figure 2 show the full range of minor/major and major PPDS prevalence rates across Canada's provinces and territories, as well as the overall national averages (8.46% and 8.69%, respectively). The rates of minor/major PPDS in Canada's provinces and territories ranged from of 4.35% in Prince Edward Island to 14.05% in the territories. Similarly, the lowest rate of major PPDS was found in an Atlantic province, New Brunswick, and the highest was found in the territories (5.03% and 15.90% respectively). Figure 3 illustrates the distribution of the total prevalence rate of PPDS among Canada's provinces and territories, with the highest prevalence rates found in the Territories (29.95%) and the lowest found in Prince Edward Island (10.89%).

Table 1 presents the estimated population and distribution of predictors of PPDS and the unadjusted associations between minor/major and major PPDS and potential predictors. Table 2 shows the adjusted associations between minor/major and major PPDS and potential predictors. Total household income, when compared to the highest total household income group, was the only significant socioeconomic variable for minor/major PPDS and major PPDS. After adjusting for the other independent variables, a prior diagnosis of depression or having been previously prescribed antidepressants remained significant for minor/major and major PPDS (OR: 1.70, 95% CI: 1.32-2.19 and OR: 2.50, 95% CI: 1.91-3.28 respectively). Interestingly, smoking status during the 3^rd ^trimester was negatively associated with experiencing minor/major PPDS after adjusting for all other variables (OR: 0.78, 95% CI: 0.66-0.91). A mother's stress level during pregnancy showed the strongest association with both minor/major and major PPDS. The odds ratios for minor/major PPDS when being "very stressed" and "somewhat stressed" were 3.59 (95% CI: 2.58-5.00) and 2.39 (95% CI: 1.86-3.09) respectively as compared to not being stressed at all. The association was even stronger between the levels of stress and major PPDS, where mothers who were "very stressed" were 6.98 (95% CI: 4.99-9.77) times more likely to experience major PPDS and those who were "somewhat stressed" were 2.28 (95% CI: 1.71-3.05) times more likely to experience major PPDS. When analyzing the relationship between the availability of support and the development of minor/major and major PPDS, having support "none of the time" no longer remained significantly associated with minor/major PPDS after adjusting for all other variables (OR: 2.26, 95% CI: 0.89-5.71), but remained significant for major PPDS (OR: 3.75, 95% CI: 1.69-8.35). Having support "some of the time" maintained a significant association with both minor/major and major PPDS (OR: 2.93, 95% CI: 2.29-3.77 and OR: 4.12, 95% CI: 3.19-5.31).

**Table 1:** Descriptive data & unadjusted analysis of potential predictors and minor/major and major PPDS

	Total		PPDS (minor/major)		PPDS (major)	
	N*	%	OR	95% CI†	OR	95% CI†
Socioeconomic Factors						
**Maternal education level**						
Less than high school	5787	7.65	**1.79**	1.27-2.53	**2.54**	1.84-3.49
High school grad	10049	13.29	**1.49**	1.11-2.01	**1.71**	1.27-2.29
Some Post-Secondary	4609	6.09	**1.74**	1.19-2.59	1.44	0.98-2.12
Post-Secondary Diploma	28304	37.43	**1.30**	1.03-1.63	1.08	0.84-1.38
University	26872	35.54	1		1	
						
**Total household income**						
Low	2250	3.13	**1.78**	1.03-3.09	**4.49**	2.89-6.97
Low-Middle	5764	8.02	**2.85**	2.05-3.97	**3.48**	2.51-4.82
Middle	15179	21.11	**1.84**	1.40-2.41	**2.04**	1.52-2.72
Middle-Upper	24643	34.27	**1.44**	1.11-1.86	**1.40**	1.05-1.86
High	24078	33.48	1		1	
						
**Occupation during pregnancy**						
No	23554	30.92	**1.38**	1.13-1.68	**2.10**	1.72-2.56
Yes	52612	69.08	1		1	
						
Demographic Factors						
**Region of residence**						
Atlantic	4521	5.91	**0.53**	0.39-.071	**0.34**	0.25-0.47
Quebec	18333	23.96	**0.46**	0.34-0.63	**0.48**	0.37-0.64
Ontario	29688	38.80	**0.58**	0.44-0.77	**0.48**	0.37-0.63
Prairies	14569	19.04	**0.40**	0.30-0.55	**0.46**	0.34-0.61
British Columbia	8997	11.76	**0.52**	0.36-0.75	**0.41**	0.28-0.59
Territories	398	0.52	1		1	
						
**Immigration status**						
Yes	16763	22.03	**1.87**	1.51-2.32	**2.71**	2.20-3.32
No	59337	77.97	1		1	
						
**Age of infant (months)**						
9-14	1709	2.24	**0.39**	0.23-0.65	**0.43**	0.25-0.74
6-8	65102	85.27	1.04	0.79-1.39	**0.72**	0.53-0.99
5	9534	12.49	1		1	
						
Maternal Characteristics						
**Parity**						
Multiparous	41556	54.53	1.10	0.91-1.32	**1.29**	1.07-1.56
Primiparous	34647	45.47	1		1	
						
**Living with a husband/partner**						
No	6374	8.36	**1.55**	1.16-2.09	**1.90**	1.46-2.49
Yes	69832	91.64	1		1	
						
**Maternal age**						
15-19	1503	1.96	**1.71**	1.05-2.78	**2.71**	1.80-4.08
20-24	8923	11.66	1.13	0.85-1.51	**1.38**	1.06-1.81
25-34	12927	16.90	1		1	
35-39	2963	3.87	**1.32**	1.04-1.68	**1.33**	1.05-1.69
40+	50192	66.37	1.01	0.58-1.77	1.41	0.87-2.27
						
**Pregnancy weight gain guidelines**						
Inadequate	24260	32.29	0.88	0.65-1.19	0.82	0.62-1.08
Recommended	13947	18.56	1		1	
Excessive	36936	49.15	1.14	0.88-1.48	0.91	0.70-1.18
						
**Previous diagnosis of depression/prescription antidepressants**						
Yes	11784	15.46	**2.05**	1.64-2.54	**2.47**	1.99-3.06
No	64456	84.54	1		1	
						
**Smoking status during 3rd trimester**						
Smoked	8016	10.50	**1.79**	1.39-2.32	**1.57**	1.22-2.04
Did not smoke	68319	89.50	1		1	
						
**Mother's stress level during pregnancy**						
Very stressed	9488	12.45	**3.91**	2.92-5.23	**7.13**	5.47-9.28
Somewhat stressed	33968	44.58	**2.64**	2.08-3.34	**2.20**	1.75-2.78
Not at all	32742	42.97	1		1	
						
**Breastfeeding initiation**						
No	7394	9.67	1.10	0.84-1.44	1.20	0.61-1.14
Yes	69086	90.33	1		1	
						
Other variables						
**Availability of support after pregnancy**						
None of the time	1095	1.44	**4.27**	2.22-8.20	**7.11**	4.08-12.41
Some of the time	11126	14.58	**3.57**	2.86-4.44	**5.75**	4.69-7.06
Most of the time	64076	83.98	1		1	
						
**Planned pregnancy**						
No	5378	7.08	1.32	0.93-1.88	**1.93**	1.43-2.61
Yes	70571	92.92	1		1	
						
**Baby in NICU**						
Yes	9733	12.74	**1.30**	1.01-1.68	1.10	0.84-1.44
No	66681	87.26	1		1	

* Sample size is estimated using population weights

ORs in bold are significant at an alpha of 0.05

† 95% CI were calculated using bootstrapping technique

**Table 2:** Multinomial logistic regression analysis of minor/major and major PPDS

	PPDS (minor/major)		PPDS (major)	
	OR	95% CI†	OR	95% CI†
Socioeconomic Factors				
**Maternal education level**				
Less than High School	1.16	0.71-1.90	1.40	0.85-2.32
High School Graduate	1.20	0.84-1.72	1.24	0.82-1.87
Some Post-Secondary	1.54	0.99-2.38	0.98	0.59-1.61
Post-Secondary Diploma	1.14	0.88-1.48	0.98	0.73-1.32
University Graduate	1		1	
				
**Total household income**				
Low	1.16	0.56-2.39	1.91	0.95-3.83
Low-Middle	**2.11**	1.39-3.20	**1.79**	1.14-2.82
Middle	**1.60**	1.16-2.21	**1.47**	1.02-2.12
Middle-Upper	**1.39**	1.05-1.85	**1.38**	1.00-1.91
High	1		1	
				
**Occupation during pregnancy**				
No	1.09	0.86-1.38	1.32	1.01-1.71
Yes	1		1	
				
Demographic Factors				
**Region of residence**				
Atlantic	1.39	0.64-3.03	1.05	0.40-2.75
Quebec	1.33	0.62-2.87	1.28	0.51-3.21
Ontario	1.37	0.62-3.01	0.98	0.39-2.46
Prairies	1.08	0.49-2.34	0.96	0.38-2.40
British Columbia	1.25	0.56-2.80	0.86	0.33-2.27
Territories	1		1	
				
**Immigration status**				
Yes	**1.84**	1.41-2.40	**2.35**	1.77-3.13
No	1		1	
				
**Age of infant (months)**				
9-14	**0.39**	0.19-0.81	0.52	0.22-1.26
6-8	1.10	0.80-1.51	0.78	0.54-1.12
5	1		1	
				
Maternal Characteristics				
**Parity**				
Multiparous	0.91	0.72-1.14	1.06	0.83-1.37
Primiparous	1		1	
				
**Living with a husband/partner**				
No	1.01	0.67-1.53	1.09	0.72-1.65
Yes	1		1	
				
**Maternal age**				
15-19	1.17	0.58-2.35	**2.03**	1.09-3.78
20-24	0.86	0.61-1.22	1.09	0.75-1.58
25-34	1		1	
35-39	1.13	0.85-1.50	1.10	0.79-1.51
40+	0.83	0.45-1.54	0.94	0.51-1.73
				
**Pregnancy weight gain guidelines**				
Inadequate	1.05	0.76-1.45	1.11	0.79-1.57
Recommended	1		1	
Excessive	1.22	0.93-1.62	1.17	0.85-1.61
				
**Previous diagnosis of depression/prescription antidepressants**				
Yes	**1.70**	1.32-2.19	**2.50**	1.91-3.28
No	1		1	
				
**Smoking status during 3rd trimester**				
Smoked	**0.78**	0.66-0.91	0.90	0.76-1.07
Did not smoke	1		1	
				
**Mother's stress level during pregnancy**				
Very stressed	**3.59**	2.58-5.00	**6.98**	4.99-9.77
Somewhat stressed	**2.39**	1.86-3.09	**2.28**	1.71-3.05
Not at all	1		1	
				
**Breastfeeding initiation**				
No	0.99	0.71-1.38	0.70	0.46-1.07
Yes	1		1	
				
Other variables				
**Availability of support after pregnancy**				
None of the time	2.26	0.89-5.71	**3.75**	1.69-8.35
Some of the time	**2.93**	2.29-3.77	**4.12**	3.19-5.31
Most of the time	1		1	
				
**Planned pregnancy**				
No	0.89	0.59-1.34	0.99	0.66-1.49
Yes	1		1	
				
**Baby in NICU**				
Yes	1.14	0.84-1.55	0.99	0.72-1.38
No	1		1	

† 95% CI were calculated using bootstrapping technique

ORs in bold are significant at an alpha of 0.05

The multinomial regression model included all independent variables listed in the table.

## Discussion

This study investigated the prevalence and characteristics of minor/major and major PPDS among mothers in the Canadian provinces and territories. The national prevalence rates of minor/major PPDS and major PPDS were found to be 8.46% and 8.69% respectively. The analysis revealed an association between total household income and both minor/major and major PPDS, which was found to decrease as household income increased. Immigration status, delivery at a young age, and a prior diagnosis of depression were all found to be positively associated with both forms of PPDS. Yet, the amount of stress during pregnancy and the lack of availability of support postpartum had the highest direct association with both minor/major and major PPDS.

This study found the national prevalence rate for major PPDS to be lower (8.69%) compared to a previous report conducted by Statistics Canada in 1998 (10-15%) [1]. The lower national prevalence rate for major PPDS may partially be attributed to the timing of the EPDS survey in this study, which was administered 5 to 14 months postpartum. A meta-analysis that analyzed several international studies found, at 6 weeks postpartum, the mean American prevalence rate of PPD to be 15.4% and the mean prevalence rate in the United Kingdom to be 12.8% [12]. The higher prevalence rate of PPDS observed in the Canadian territories may partially be attributed to the population being comprised of a greater proportion of aboriginal people. Studies have shown that aboriginal people are at increased risk of suffering from depression [31]. The low minor/major and major PPDS prevalence rates found in the Atlantic provinces may be related to the reported high levels of social support [32].

The association between total household income and both minor/major and major PPDS were found to be higher with a decrease in lower income group, which has been seen previously in the literature. A meta-analysis based on 59 studies, also found that a decreased household income was associated with a greater risk for PPD [2]. This may partly be attributed to the increased amount of stress placed on a mother due to the availability of limited financial means necessary for raising an infant [2,26]. Variation in the external environment (urban/rural) in conjunction with socioeconomic status may also affect the association found.

Immigrants were at increased odds of experiencing minor/major PPDS (OR: 1.84, 95% CI: 1.41-2.40), and major PPDS (OR: 2.35, 95% CI: 1.77-3.13) compared to non-immigrants. Consistent with this finding, a Canadian study conducted with participants from the Calgary Health Region between 2001 and 2004, report that having been born outside of Canada was associated with an increased risk of 1.87 of developing minor/major PPD (95% CI: 1.17-3.00) [6]. However, such an association may have been moderated by time since immigration, which may account for the effects of acculturation that could be present among immigrants who have spent substantial periods of time in Canada. The added stresses that accompany living in new surroundings among an unfamiliar culture, may compound the pressures that coincide with being a parent of a newborn.

Within the maternal characteristics, mothers between the ages of 15 and 19 years, a prior diagnosis of depression or past use of prescription antidepressants, smoking during the 3^rd ^trimester, and a mother's stress level during pregnancy were all associated with experiencing PPDS. Although adolescent mothers were found to be associated with major PPDS (OR: 2.03, 95% CI: 1.09-3.78), the literature is not conclusive when it comes to the association between maternal age and PPD; subsequently it is regarded as a possible predictor of PPD [2,5]. However, concerning the adolescent population, higher prevalence rates of PPD have been reported [5,7,33].

A prior diagnosis of depression or past use of prescription antidepressants was associated with a higher odds of experiencing both minor/major and major PPDS. This substantial higher risk of PPDS is in concordance with previous literature regarding depression history independent of childbirth [2,21,34]. A mother's stress level during pregnancy was significantly and substantially associated with experiencing symptoms of both minor/major and major PPDS. The current literature is in agreement with the findings of the present study [2,5,35]. Although occupation during pregnancy, living with a husband/partner, and planned pregnancy did not remain significant in the adjusted model, their relationship with PPDS may have been partially accounted for with the significantly higher associations observed between stress and the risk of experiencing minor/major and major PPDS (OR: 3.59, 95% CI: 2.58-5.00 and 6.98, 95% CI: 4.99-9.77 respectively). Accompanying the significant results for stress during pregnancy are significant results regarding the amount of support available to the mother postpartum. Using the referent category "most of the time" for the variable of support after pregnancy garnered a significantly stronger positive relationship with minor/major and major PPDS when support was available "some of the time" compared to "none of the time". This association is seen when a lack of necessary social support is present in the form of family and friends, as well as professionals [26,36].

This was the first national study that analyzed the national, provincial, and territorial prevalence rates of minor/major and major PPDS. An extensive list of characteristics of PPDS was examined among a diverse and representative sample of Canadian women. Conducting this study with a large sample size increased the statistical power. The current study was based on a cross-sectional survey. Information bias may be present due to the self-report nature of the MES. A few of the characteristics assessed may be affected by recall bias, such as a mother's stress level during pregnancy, whether she adhered to the pregnancy weight gain guidelines, and recalling a past history of depression and/or treatment with antidepressants. However, recall bias was limited for the measurement of PPDS, due to the fact that the EPDS refers to the past seven days. A limitation of the study was the timing of the administration of the EPDS. Since the criteria for the date of the births was different for the provinces and the territories, it created a sampling period that ranged from 5 to 14 months postpartum, which ultimately garnered conservative minor/major and major PPDS prevalence rates. Although symptoms of PPD can last up to 14 months [5], there is a chance that lower prevalence rates were observed in our study, due to the fact that symptoms of PPD may have resolved by the time the participants were surveyed. In an attempt to account for this, the adjusted model controlled for the age of the infant. As well, since the MES was conducted during the winter months in the territories, seasonal variations in prevalence of PPDS may have been present [37], which may have inflated the prevalence rates seen in the territories. The fact that a confirmatory instrument was not used in this study presents a limitation; hence the results of the study refer to postpartum depression symptomatology. A lack of evidence regarding the validity of the EPDS when administered by telephone, may result in a misclassification bias. Another factor that may have affected the results of this study was the differences between the respondents and the non-respondents. However, in order to decrease the non-response bias, weighting adjustments designed by Statistics Canada were applied to all variables in the MES. Information about the mothers' knowledge of symptoms of PPD prior to delivery, as well as knowledge of various treatment options and accessibility to them, would have benefited the current study.

## Conclusions

Information from this study can aid health practitioners in addressing at-risk groups and inform prospective mothers of signs of PPDS, in order to increase both the timely attainment of treatment (i.e. antidepressants, professional psychotherapy, telephone based peer support) and the rate at which it can be obtained. Interventions that would specifically target women with a prior history of depression, less than adequate social support, are immigrant or adolescent mothers, or are experiencing high levels of stress may help to decrease the prevalence of PPDS among these populations.

## Competing interests

The authors declare that they have no competing interests.

## Authors' contributions

AL performed the analysis and the write up for the manuscript. JK consulted on the manuscript. HT supervised the analysis and the write up of the manuscript. All authors read and approved the final manuscript.

## Pre-publication history

The pre-publication history for this paper can be accessed here:

http://www.biomedcentral.com/1471-2458/11/302/prepub
